# An Alternative Chemical Redox Method for the Production of Bispecific Antibodies: Implication in Rapid Detection of Food Borne Pathogens

**DOI:** 10.1371/journal.pone.0091255

**Published:** 2014-03-17

**Authors:** Mohammad Owais, Shadab Kazmi, Saba Tufail, Swaleha Zubair

**Affiliations:** 1 Interdisciplinary Biotechnology Unit, Aligarh Muslim University, Aligarh, India; 2 Women's College, Aligarh Muslim University, Aligarh, India; Indian Institute of Science, India

## Abstract

Bi-functional antibodies with the ability to bind two unrelated epitopes have remarkable potential in diagnostic and bio-sensing applications. In the present study, bispecific antibodies that recognize human red blood cell (RBC) and the food borne pathogen *Listeria monocytogenes* (*L. monocytogenes*) were engineered. The procedure involves initial reduction of a mixture of anti-RBC and anti-Listeria antibodies followed by gradual re-oxidation of the reduced disulphides. This facilitates association of the separated antibody chains and formation of hybrid immunoglobulins with affinity for the *L. monocytogenes* and human RBC. The bispecific antibodies caused the agglutination of the RBCs only in the presence of *L. monocytogenes* cells. The agglutination process necessitated the specific presence of *L. monocytogenes* and the red colored clumps formed were readily visible with naked eyes. The RBC agglutination assay described here provides a remarkably simple approach for the rapid and highly specific screening of various pathogens in their biological niches.

## Introduction

Bispecific antibodies (BsAbs) are crafted using either intact antibodies or their fragments such as single domain antibodies, Fabs (fragments antigen binding) and scFvs (single-chain fragments variable) [1]. The unique ability of BsAbs to bind two distinct epitopes simultaneously is the hallmark of their therapeutic potential. Additionally, BsAbs obtained by fusing two or more antibodies which bind different epitopes on the same antigen have been traditionally used for increasing the avidity of antigen antibody interaction [2], [3]. BsAbs prepared by fusing an antibody specific for an effector cell to a target cell-specific second antibody have also been used for activating innate and adaptive immune responses of the host [3], [4]. In spite of the robustness of the fabricated antibody based strategies, potential of BsAbs has not been fully explored either in bio-sensing applications or detection and screening of organisms affecting health of higher animals.

Various existing methods employed for preparation of BsAbs suffer with inherent limitations; (A) Chemical cross-linking of two antibody molecules or their fragments [5] sometimes results in inactivation, unfolding or aggregation of the synthesized BsAbs [6]. (B) Fusion of two or three different cell lines to form a quadroma or trioma [7], a strategy necessitating lengthy cell culturing but usually gives poor yield of the BsAbs [8]. (C) Recombinant DNA based approach involving cloning and expression of single chain variable fragment (scFv) fusions or diabodies, scFv-Fc fusions and single variable domain IgGs as well as dual-variable domain IgG [9]–[12]. The procedure entails good technical expertise and sophisticated instrumentation.

For detection and screening of various pathogens, techniques based on cell culture, PCR and immuno-assays are widely employed [13]. While these strategies are highly sensitive as well as consistent, they unfortunately are time consuming and expensive. The large number of outbreaks of infections globally, and illnesses they manifest, advocate the need for simple and rapid procedures for identification of the causative pathogen. Annually, on an average about 1–2 million people are estimated to be infected by bacteria, of which 70% are food borne [14], [15]. Addressing a problem of such magnitude, especially in the under developed and poor countries is possible only if simple, rapid and inexpensive diagnostic tools for detection of various pathogens become available.

We describe in this article a strategy employing BsAbs for the specific and rapid detection of *L. monocytogenes* in food and other biological samples. BsAbs recognizing the cell surface antigens of human erythrocytes and *L. monocytogenes* were generated using a modified reduction/oxidation procedure [5]. The hybrid antibodies induced the agglutination of human erythrocytes specifically in the presence of *L. monocytogenes* cells and the resulting red cell clumps were large enough to be visible to the naked eye. BsAbs prepared both from monoclonal as well as polyclonal antibodies were equally effective in inducing the agglutination.

BsAbs were successfully employed for the highly precise, specific and ready detection of *L. monocytogenes*. This inexpensive but robust sensing technique neither requires sophisticated instrumentation nor any special skill and can be used in any modest clinical laboratory. *L. monocytogenes* was chosen for the study, since listeriosis is most widespread amongst various food-borne pathogens and leads to very high fatality rate (25%–30%) [16]. The *L. monocytogenes* contamination has prompted imposition of zero tolerance limits by U.S. regulatory agencies [17], [7]. Unfortunately, the detection methods currently in vogue require sophisticated instrumentation and are slow needing a time lapse (at least 24 hrs) to deliver concrete information [7].

## Materials and Methods

### Chemicals and reagents

All the chemicals and reagents used were of the highest purity available. Phenylmethanesulfonylfluoride, β–mercaptoethanesulfonic acid sodium salt, β–mercaptoethanol, Ethanolamine, Sepharose-4B, Freund's complete and incomplete adjuvant, Bicinchoninic acid (BCA) protein estimation kit, Tween-20 and FITC conjugated mouse anti-rabbit antibody were purchased from Sigma-Aldrich Chemicals (St Louis, MO) and used as received. Horseradish peroxidase-conjugated anti-rabbit IgG were purchased from Bangalore Genei (India) Pvt. Ltd. (Bangalore, India). Mouse monoclonal anti-Listeria LZH1 IgG1 and mouse monoclonal IgG2a specific for the human erythrocyte membrane protein (Protein4.2 (2G-12)) were procured from Santa Cruz Biotechnology, Inc., California and Abnova Corporation respectively.

### Ethics statement

The study was approved by the Institutional Animal Ethics Committee of the Interdisciplinary Biotechnology Unit, Aligarh Muslim University, Aligarh, India. All animal experiments were performed according to the National Regulatory Guidelines issued by the Committee for the Purpose of Control and Supervision of Experiments on Animals (CPCSEA). Our approval ID was 332/CPCSEA, Ministry of Environment and Forests, Paryavaran Bhavan, Government of India.

### Animals

Inbred female rabbits, 8–10 weeks old, were obtained from the Department Animal House Facility, Interdisciplinary Biotechnology Unit, AMU, India. The animals were kept on standard pellet diet and water *ad libitum*. All the rabbits were housed under standard conditions at the Department Animal House Facility.

### Blood Collection

The study was conducted on discarded blood procured from Blood Bank, Department of Pathology (R. No.-BBL-04/SC/P/1996), Jawaharlal Nehru Medical College, Aligarh Muslim University, Aligarh, India.

### Preparation of erythrocyte ghost

The erythrocyte ghosts were prepared using published procedure as standardized in our laboratory [18]. Briefly, whole blood (human) was mixed with anticoagulant solution (EDTA, 2.7%) and centrifuged at 1500 g for 10 minutes. Buffy coat was discarded and the RBC pellet was washed with 20 mM phosphate buffered saline, PBS (pH 7.4). The RBC pellet was lysed with chilled hypotonic buffer (5 mM Tris-HCl, pH 7.01) in 1∶40 ratio. This suspension was stirred at 4°C for 1 hour. The hemolysed suspension was centrifuged at 10,000 g to get the lysed erythrocytes. The pellet was washed with chilled hypotonic buffer until a white pellet was obtained. To initiate vesiculation (resealing), each ml of packed unsealed ghost was diluted to 40 ml with ice cold 20 mM PB in the presence of 1 mM MgSO_4_ solution and stirred for 2 hours at 4°C. The suspension was pelleted at 10,000 g and washed twice with the same buffer [18], [19]. Membrane proteins associated with erythrocyte ghost were analysed in 10% sodium dodecyl sulphate-polyacrylamide gel electrophoresis (SDS-PAGE).

### Preparation of antigen from *Listeria monocytogenes*

*L. monocytogenes* (ATCC 15313) was procured from ATCC (American Type Culture Collection, Manassas, VA). InlB protein of *L. monocytogenes* was purified using the protocol described elsewhere [20], [21]. Briefly, *L. monocytogenes* was cultured on Brain Heart Infusion broth medium at 37°C overnight. The exponentially growing bacterial cells were harvested at 5000 g for 10 minutes and washed with 150 mM Normal Saline twice. The pelleted bacteria were immediately re-suspended in approx. 0.5% of the original culture volume using 1M Tris-HCl buffer (pH 8). Re-suspended bacteria were incubated for 60 min on ice. The bacterial suspension was pelleted at 12000 g for 20 minutes and the supernatant was collected [20], [21]. The concentration of the purified protein was determined by the BCA method of protein estimation [22].

### Generation of affinity columns

Cross-linked erythrocyte stroma was prepared to isolate anti-RBC and in-house prepared bispecific antibodies. We followed published procedure for the preparation of cross-linked stroma [23], [24]. Briefly, blood (50 ml) was centrifuged at 1500 g. Supernatant along with buffy coat was removed and cells were washed three times with normal saline. A 10% suspension of erythrocytes was prepared in 5 mM hypotonic phosphate buffer (50 ml) and mixed with digitonin (250 μl, 5 mg/ml) with stirring at 4°C. It was centrifuged at 12,000 g for 10 minutes at 0°C. The pellet was thoroughly washed with normal saline till the supernatant was free from protein as determined by absorbance at 280 nm. About 4 g of stroma was obtained from 50 ml of blood. The stroma, thus prepared, was washed two times with 10 mM Tris buffer (pH 7.4) and 10 mg/ml (wet weight) was suspended in the same buffer. It was mixed with an equal volume of 6.25% solution of glutaraldehyde. The mixture was stirred for 4.5 hours at 37°C followed by stirring at 4°C for 12 hours. All the operations were carried out in dark. After stipulated time period, the stroma was centrifuged at 12,000 g for 10 mins at 4°C to obtain pellet. This pellet was washed with normal saline till the supernatant was free of any protein.

Cyanogen bromide activated Sepharose 4B was used to prepare the affinity column of a *Listeria monocytogenes* cell surface protein, InlB [25]. Briefly, 10 g Sepharose 4B was washed with distilled water followed by the addition of 2 M Na_2_CO_3_ solution. Further, 1 g CNBr in acetonitrile was added with constant stirring. After 5 mins, the activated gel was washed with the coupling buffer (0.1 M NaHCO3, 0.5 M NaCl, pH 8.5). Gel was suspended in the coupling buffer containing InlB protein. Unbound protein was quantitated in washed out elutes. Remaining active groups were blocked using excess of ethanolamine for 2 hours at room temperature.

### Production of anti-RBC antibody

Primary immunization was executed by immunizing female rabbit with 150 μg (wet weight) of RBC ghost in combination with complete Freund's adjuvant. The first booster was given with 100 μg of RBC ghost in incomplete Freund's adjuvant emulsion after 21 days of first immunization. The second booster was administered after 28 days of primary immunization. On the fifth day of second booster, animals were bled and sera were collected. RBC surface antigen specific antibodies were purified from sera using affinity column of cross-linked erythrocyte stroma to obtain antibodies specific for RBCs. Briefly, sera were incubated with the cross-linked stroma to allow specific interaction and then RBC stroma was washed extensively with PBS (pH 7.4) to remove unbound non-specific antibodies. Bound antibodies were eluted using 0.1 M Glycine-HCl, pH 2.7 and the pH of elute was neutralized by the addition of a 1/10 dilution of 1.0 M Tris-HCl, pH 9.0. The eluted fraction from the RBC stroma was dialyzed against three changes of PBS (pH 7.4).

### Immunization protocol for the development of *Listeria* cell surface protein specific antibody

Female rabbits were immunized with *Listeria* InlB cell surface protein (100 μg) emulsified with complete Freund's adjuvant. The booster dose (50 μg antigen) was administered on day 21 post primary immunization. On the fifth day of the booster, blood was collected to obtain sera. Antibodies were purified from sera using the in-house prepared *Listeria* affinity column to obtain specifically anti-*Listeria* antibodies. Antibodies bound to the *Listeria* affinity column were eluted using 0.1 M Glycine-HCl, pH 2.7 and the pH of elute was neutralized by the addition of a 1/10 dilution of 1.0 M Tris-HCl, pH 9.0. The eluted fraction from the *Listeria* affinity column was dialyzed against three changes of PBS (pH 7.4).

### Construction of BsAb employing Redox methodology

**Figure 1:**
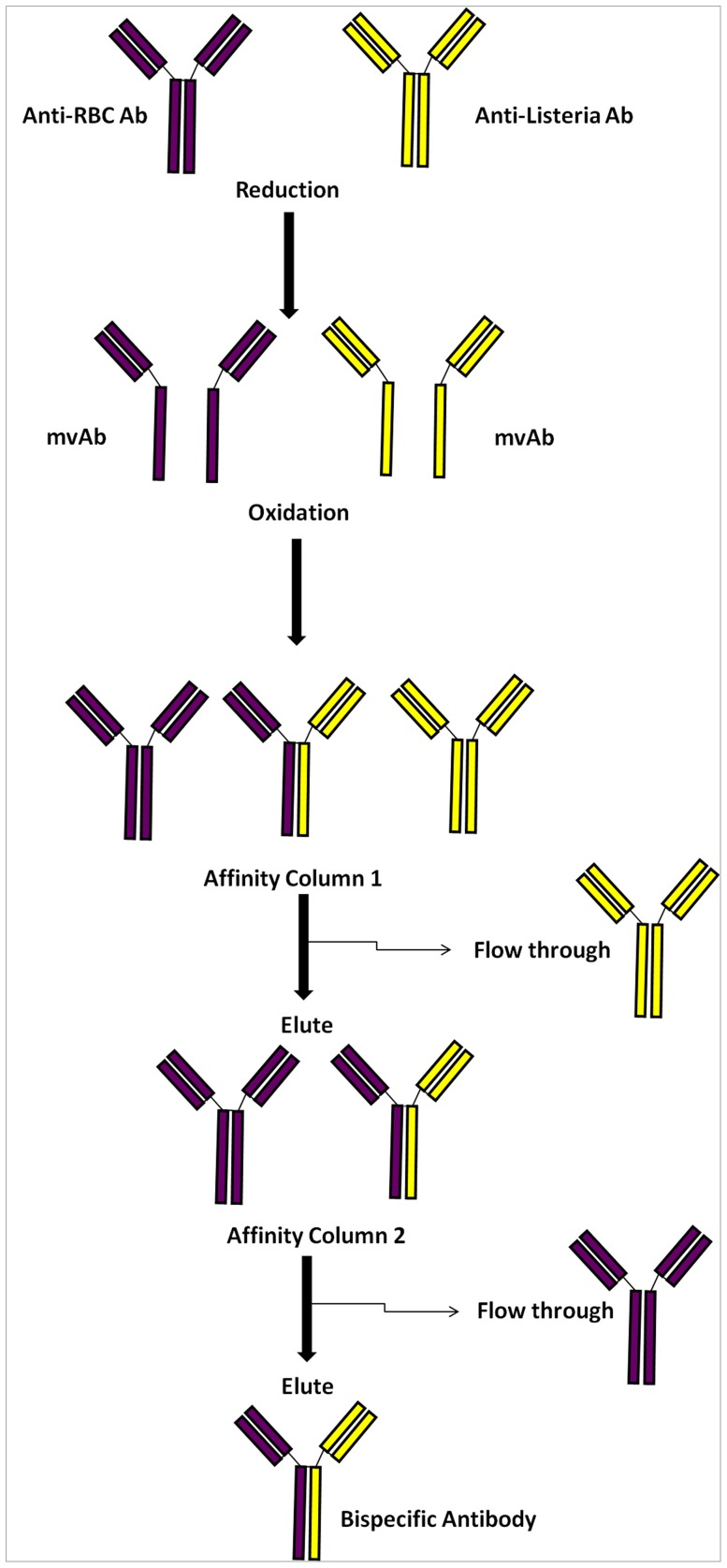
Schematic representation of production and purification of Bispecific antibody (BsAb). Anti-RBC antibody and anti-listeria antibody are mixed and reduced using reducing agent, resulting in mvAb fragments. When dialysed into oxidising conditions antibodies are reformed resulting in a mixture of BsAb of different parent lineage. The resulting elute, after affinity column 1 and 2, contains pure BsAb specific for both RBC and *Listeria* surface antigens.

The redox method was employed for the preparation of bispecific antibodies. A schematic representation of the redox method used for BsAb production is shown in **Figure 1**. Increasing concentrations (0–60 mM) of reducing agents, viz. β–mercaptoethanesulfonic acid sodium salt and β–mercaptoethanol were analyzed for efficient reduction of the disulphide bonds of both monoclonals as well as polyclonals antibodies (data not shown for β–mercaptoethanol). The monoclonal antibodies were reduced by adding an equal volume of two fold concentrated β-mercaptoethanesulphonic acid sodium salt solution (60 mM) in distilled water to a mixture of 1 mg of mouse monoclonal anti-*L. monocytogenes* LZH1 IgG1 and mouse monoclonal IgG2a raised against the human erythrocyte membrane protein (Protein4.2 (2G-12)) in PBS and incubated at 37°C for 25 minutes. Thereafter, monovalent arms were exposed to oxidizing conditions by dialysis against three buffer exchanges of phosphate buffered saline (PBS), pH 7.4 for 24 hours at 4°C [9]. The same set of protocol was followed for the construction of the in-house prepared bispecific antibody, employing polyclonal antibodies specific for human erythrocyte or *L. monocytogenes* cell surface antigens. Bispecific antibodies from both sources (monoclonal and polyclonal) were purified from parent antibody mixtures over sequential affinity columns (as discussed in detail later) to ensure that only pure and surface antigen specific BsAbs were isolated.

### Hybrid BsAb purification

BsAbs from both the sources (monoclonal as well as polyclonal) were purified from parent antibodies using cross-linked erythrocyte stroma. The bound antibody was eluted using 0.1 M Glycine-HCl, pH 2.7 and the pH of elute was neutralized by the addition of a 1/10 dilution of 1.0 M Tris-HCl, pH 9.0. The eluted fraction from the RBC stroma was dialyzed against three changes of PBS and subsequently purified over affinity purified *L. monocytogenes* membrane proteins. The bound BsAbs were eluted using 0.1 M Glycine-HCl, pH 9.0 and subsequently extensively dialysed against PBS [9].

### Binding of hybrid BsAbs to *L. monocytogenes* cell surface proteins and human red blood cell surface antigens

The specificity of in-house prepared mBsAbs as well as pBsAbs was ascertained by studying its interaction with specific antigens. To check the binding efficacy of BsAb to Listeria and RBC surface antigens, ELISA was performed. Firstly, the plates were coated with RBC antigens. The antigen-coated plates were washed with PBS and blocked with 200 μl per well of 1% bovine serum albumin (BSA) in PBS at 37°C for 4 hour. Bispecific antibody (1∶2000 dilution) was dispensed (100 μl per well) and the plate was then incubated at 37°C for another 2 hours, followed by washing three times with PBS containing 0.05% Tween-20. The plates were further incubated with *Listeria* antigen InlB. After usual washing steps, the plates were incubated with anti-InlB antibodies raised in rabbit. After excessive washing of the plates, 100 μl of (1∶5000 dilution of stock) HRP conjugated goat anti-rabbit IgG antibodies (3° antibody) were added to the wells. The plates were further incubated at 37°C for 1 hour. The plates were washed again before the addition of 100 μl of substrate solution (6 mg O-phenylenediamine dihydrochloride (OPD) salt dissolved in 12 ml of substrate buffer with 5 μl of 30% H_2_O_2_) and were finally incubated at 37°C for 40 minutes. The reaction was terminated by the addition of 50 μl of 7% H_2_SO_4_. The absorbance was read at 490 nm with a microtitre plate reader (Bio-Rad, Hercules, CA 94547 USA). Similarly, the specificity of bispecific antibodies was also tested by coating *Listeria* antigen, InlB on the plate and detecting the signal using anti-RBC antibodies raised in rabbit. Briefly, BsAbs pre-incubated with erythrocyte antigens were used as primary antibody. After routine washing steps, the wells were exposed to anti erythrocyte antibodies. Next, plate was incubated with HRP conjugated goat anti rabbit and color was developed using routine protocol.

### Western Blot Assay

Western blot analysis was used to study the interaction of mBsAbs as well as pBsAbs with RBC ghost proteins and *Listeria* cell surface proteins. Briefly, RBC ghost proteins (50 μg) were resolved by electrophoresis on 10% sodium dodecyl sulfate polyacrylamide gel and then electroblotted onto PVDF membrane. The blot was blocked overnight with 3% nonfat dry milk and probed with BsAbs at 1∶1000 dilution. After stipulated incubation, the strips were washed thrice in PBST and further incubated for 1 hr at 37°C with horseradish peroxidase-conjugated mouse anti-rabbit antibody (1∶5000). The strips were washed with PBST three times and finally the immunoblots were developed on X-ray film by enhanced chemiluminiscence (ECL) using ECL kit, BioRad. Western blot of *Listeria* membrane proteins (30 μg) was also analyzed, following the same method described as above.

### Dot blot assay

Listeria and RBC antigens were dispensed onto PVDF strips carefully and allowed to dry at room temperature. The strips were rinsed briefly in phosphate-buffered saline (PBS; pH 7.4) containing Tween-20 (PBST) and were incubated overnight at 4°C in 5% non-fat dry milk in PBST to block the residual binding sites on the paper. The strips were rinsed three times in PBST. The antigen coated strips were incubated with BsAb of 1∶1000 dilution. Immunoreactive dots were detected by horseradish peroxidase-conjugated mouse anti-rabbit IgG using chromogen 3,3′-diaminobenzidine tetrahydrochloride.

### Interaction of RBCs and *Listeria monocytogenes* cells with BsAb

Interaction of BsAb (of both monoclonal and polyclonal origin) with RBCs as well as *L. monocytogenes* surface proteins was further assessed using fluorescence microscopy. RBCs (20 μl from 60% hematocrit stock) were mixed with 10^3^ *Listeria* cells in the presence of BsAbs. After agglutination, the cells were incubated with FITC conjugated mouse anti-rabbit antibody for 30 minutes and washed with PBS and finally transferred onto a glass slide to be analyzed under fluorescence microscope [26].

### Fluorescence activated cell sorter based binding analysis

FACS analysis was performed using the procedure described elsewhere standardized as per requirement of the experiment [26]. Briefly, *L. monocytogenes* cells (1×10^6^) were incubated with in-house prepared BsAb (1 μg/ml) for 30 minutes at 4°C and washed with dilution buffer (0.01 M phosphate buffer saline, pH 7.4 containing 1% bovine serum albumin, and 0.1% sodium azide) three times and finally re-suspended in 500 μl of 2% paraformaldehyde. FITC-tagged anti-rabbit secondary IgG was used as detection antibody. The percentage of positive cells were measured at various time intervals using fluorescence activated cell sorter (Easycyte Mini; Guava Technologies, Hayward, CA), and the data were analyzed accordingly.

### Agglutination assay

In the RBC and *Listeria* agglutination test, 10^3^ *Listeria* cells were mixed with 20 μl of human erythrocyte (60% hematocrit stock) and dispensed to the wells of a 96-well microtitre plate. Two fold serial dilution of BsAbs (poly/monoclonal origin) was made before mixing RBC and *Listeria* cells, in 100 μl of PBS. After 10 minutes of incubation at room temperature, the mixture was scored visually to assess degree of agglutination. To confirm RBC and *Listeria* agglutination, the agglutinated mass was observed under a light microscope.

### Sensitivity analysis of agglutination assay with pure *Listeria* cultures

Overnight-grown bacterial cultures were centrifuged to harvest cell pellet and washed with PBS three times. Bacterial pellet was serially diluted in PBS to get desired number of bacterial cells. A known number of cells with density 10^2^, 10^3^, 10^5^ and 10^7^ were dispensed in various wells of microtiter plates. Subsequently, fixed number of RBCs (20 μl of 60% hematocrit) were added to each well and the mixture was finally reacted with mBsAbs as well as pBsAbs. The minimum number of Listeria cells that could induce agglutination determined the level of sensitivity.

### Detection of *Listeria monocytogenes* in contaminated food samples by agglutination assay

Beef, mutton and chicken were purchased from local grocery stores in Aligarh, U.P., India. Absence of *L. monocytogenes* in procured meat samples was confirmed by following the procedure as described elsewhere [27]. Briefly, 25 g of each meat sample was mashed and kept in sterile container. The sample was enriched using 250 ml of half-strength Fraser Broth (½ FB: Difco Lab, Sparks, MD, USA) followed by homogenisation of the meat samples for 2 min using the Stomacher 400 (Seward, Norfolk, UK). The sample homogenates were then incubated at 37°C for 18 h. Several aliquots were collected from each sample and tested by agglutination assay as described above.

For artificially contaminated samples, bacterial cell suspension with approx. density (1×10^3^ CFU) was used to inoculate 25 g of meat samples (approx. 40 CFU/g). After incubation for 15 min at room temperature to allow bacterial adaptation, enrichment step was followed as described elsewhere [27]. Ten millilitres of enriched samples was withdrawn from each sample, centrifuged (16, 000 g for 10 min), and the pellets were resuspended in 10 ml of PBS. A specific volume was used to test the presence of *L. moncytogenes* by agglutination assay.

### Statistical analysis

Statistical significance (*P* value) was ascertained by performing *t* tests on antibody titre data by using Sigma plot statistics software package (v 10 and 11; SigmaPlot, San Jose). *P* values<0.05 were considered significant.

## Results

As a proof of the concept, anti-*Listeria* mouse monoclonal LZH1 antibody specific for *Listeria* cell surface protein and mouse monoclonal antibody directed against human erythrocyte membrane protein (Protein 4.2 [2G-12]), were used in the construction of a mBsAb. The LZH1 has IgG1 isotype and recognizes cell surface protein of *L. monocytogenes*. The monoclonal antibody directed against transmembrane protein 4.2 of erythrocyte belongs to IgG2a isotype. LZH1 recognizes a 23 kDa protein of *Listeria monocytogenes* and 2G-12 is reactive to Protein 4.2 of RBCs which has mol. wt. of 72 kDa.

The specificity of hybrid BsAb consisting of one arm recognizing Listeria surface antigen and other recognizing protein 4.2 was established by its potential to induce formation of agglutinated cell mass that contained both erythrocyte and *Listeria* cells. Similarly, possibility of construction of similar hybrid antibody using polyclonal antibodies was also examined. Rabbit polyclonal antibodies raised against intact human erythrocytes in rabbits and those against InlB surface protein of *L. monocytogenes* were used for the formation of pBsAb. The mBsAb and pBsAb recognized both *L. monocytogenes* cells as well as human erythrocytes simultaneously.

### Specificity of antibody generated against RBC ghosts and *Listeria monocytogenes* surface antigen, InlB in rabbit sera

**Figure 2:**
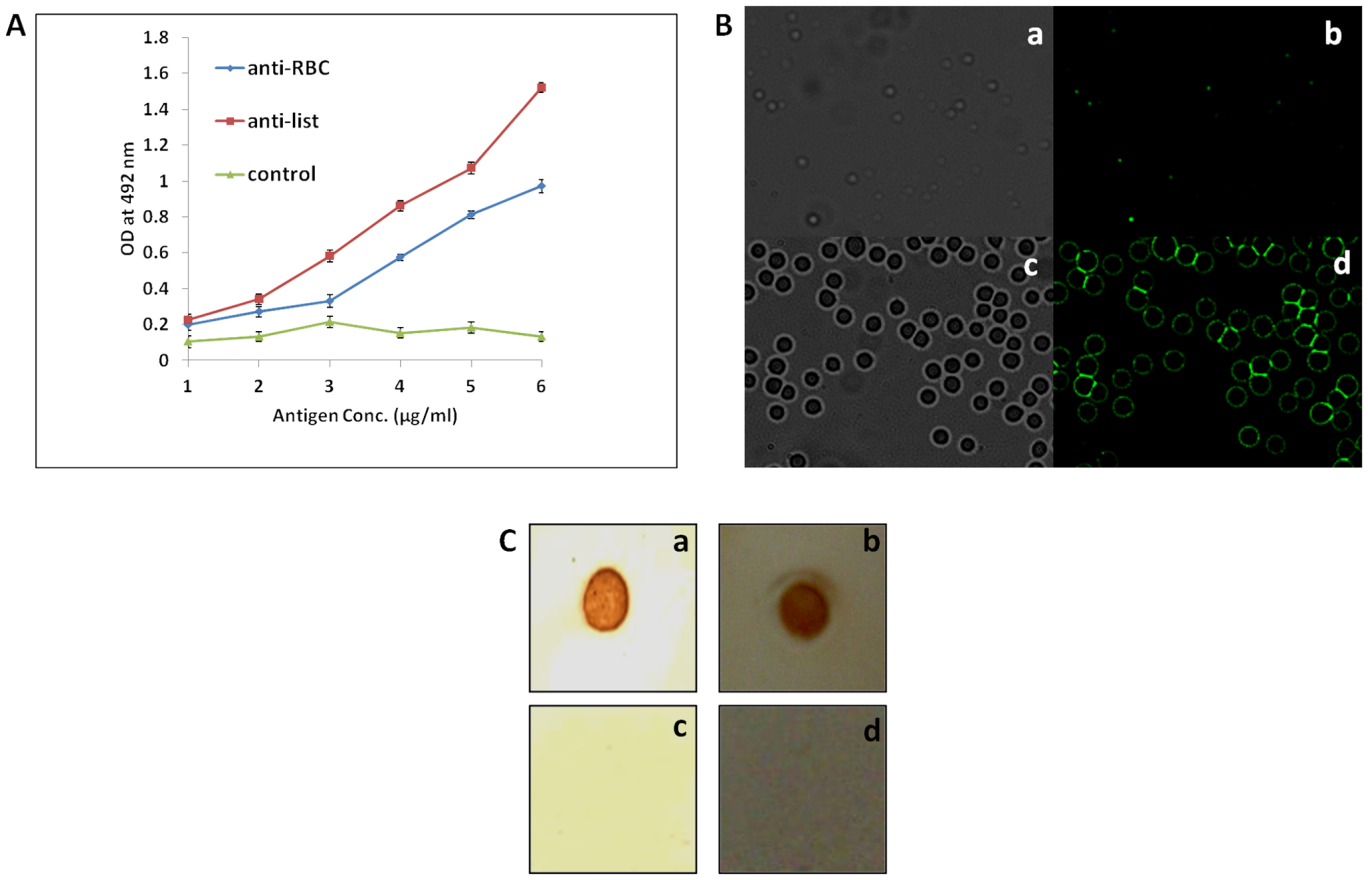
Specificity characterization of antibody generated against RBC ghosts and *Listeria monocytogenes* in rabbit sera. **A**) ELISA analysis: Antibody titer against *Listeria* surface antigens and RBC ghost proteins. Data are represented as mean ± standard error of three independent experiments (*P* values; Anti-RBC Ab vs control <0.01, Anti-List Ab vs control <0.01). **B**) Fluorescence microscopy: Anti-*Listeria* antibody interaction with cell surface proteins of *Listeria monocytogenes* (**a and b**), anti-RBC antibody binding to human RBCs (**c and d**). **C**) Dot blot analysis: **a** and **b** exhibit recognition of *Listeria* surface antigens by monospecific anti-*Listeria* antibody and interaction of RBC ghost proteins by anti-RBC antibody respectively. The controls where InlB antigen of *Listeria* (c) and RBC antigens (d) were spotted onto PVDF membrane respectively. InlB antigens were allowed to react with anti-RBC while RBC antigens were allowed to interact with anti-InlB monospeific antibodies respectively. At least three independent experiments were performed for each sample and data are representative of three independent experiments with similar observations.

Sera were isolated from the blood of immunized rabbit and assayed for anti-RBC as well as anti-*Listeria* cell surface antibodies by ELISA, fluorescence microscopy and dot blot as depicted by Figure 2. The results enumerate generation of antibodies specific for RBCs and *L. monocytogenes*. Fig. 2A demonstrates potential immunogenicity of the RBC antigens and InlB *Listeria* antigen. With increase in antigen concentration, absorbance at 492 nm was found to increase consistently and was significantly higher than controls (*P*<0.01). Fluorescence restricted to the surface of *Listeria monocytogenes* and RBCs further enumerates generation of antibodies specific for *Listeria* and RBC cell surface (Fig. 2B). The antigen-antibody interaction was validated by dot blot assay showing positive result for specificity of InlB and RBC antigens with respective antibodies developed upon their immunization (Fig. 2C). The controls exhibited negative results.

### Construction of bispecific antibodies and evaluation of their specificity by ELISA, dot blot and Western blot analyses

**Figure 3:**
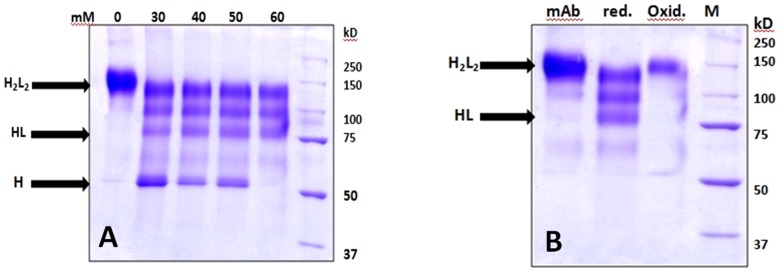
Optimisation of Redox method. Non-Reducing SDS-PAGE analysis of mvAb and BsAb of monoclonal origin. A range of β-mercaptoethanesulphonic acid concentrations were analysed for effective reduction monoclonal anti-erythrocyte antibodies. β-mercaptoethanesulphonic acid concentration of 60 mM (reducing condition) efficiently cleaved the inter- disulphide bridges between heavy chains and lead to formation of mvAb (≥75 kD) of monoclonal anti-RBC antibodies (**A**). Similarly, 60 mM of β-mercaptoethanesulphonic also rendered effective reduction of monoclonal anti-Listeria antibodies (data not shown). Dialysis against PBS (oxidizing condition) resulted in reformation of mvAb against RBC and *Listeria* into BsAb (**B**). The lanes marked mAb, red., oxid. and M in **B** enumerate anti-Listeria monoclonal antibody, reduction of anti-Listeria mAb using 60 mM of β-mercaptoethanesulphonic acid, oxidation of anti-RBC mvAb and anti-Listeria mvAb and protein marker respectively. H_2_L_2_ =  whole antibody, HL =  mvAb, H =  heavy chain, red. = reducing condition, oxid. =  oxidizing condition. At least two independent experiments were carried out for each sample and data are representative of two independent experiments with similar observations.

**Figure 4:**
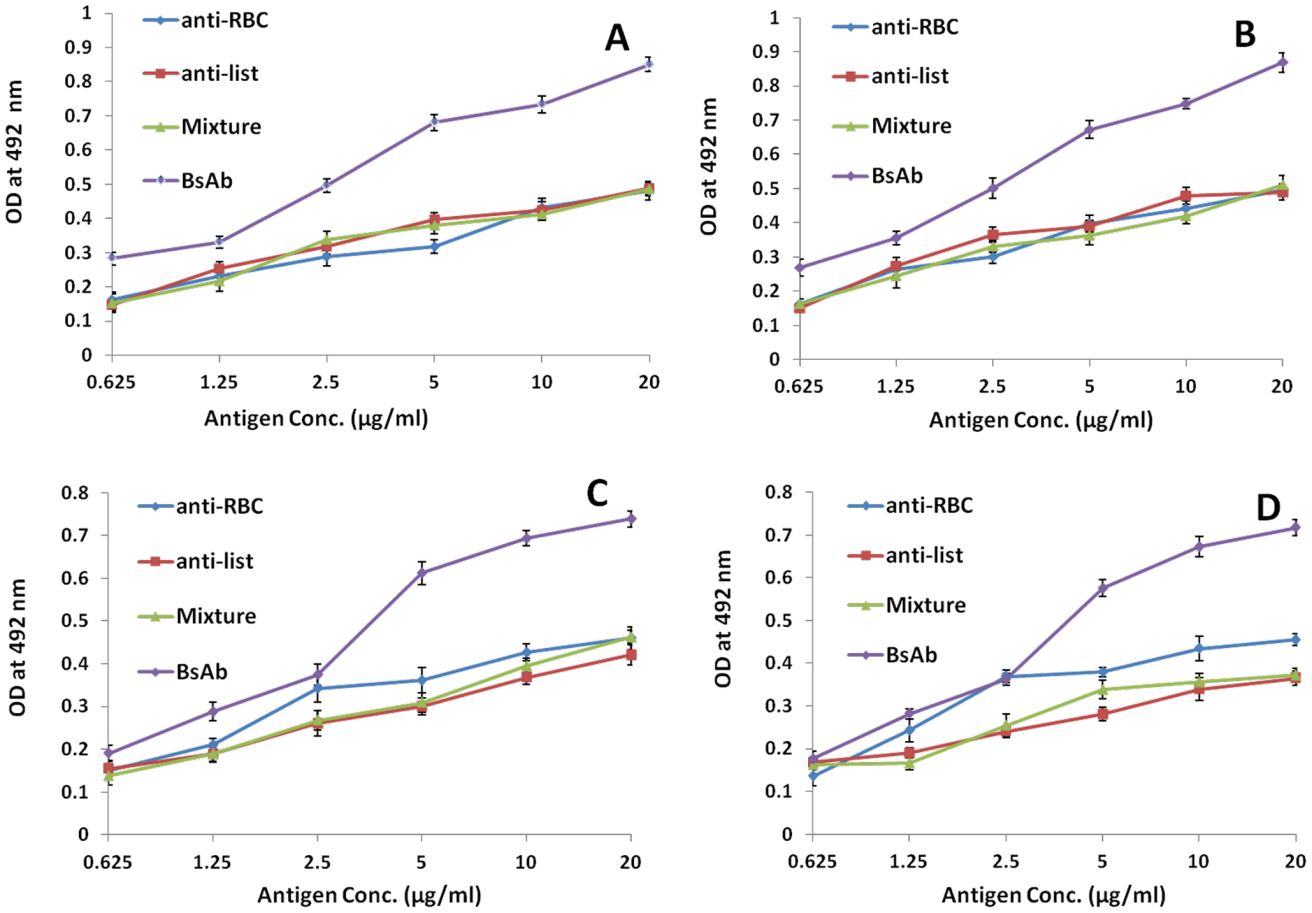
Specificity determination of in-house prepared BsAb by ELISA. Either Listeria Ag or RBC antigen was coated followed by recognition with BsAb so that its one arm was fully captured with the antigen. Further RBC Ag or Listeria Ag (which was not used for coating) was added for stipulated time period. The plate was thoroughly washed and allowed to interact with Rabbit anti RBC antibody or Rabbit anti *Listeria* antibody. Finally, the signal was detected using HRP-tagged goat anti-rabbit Ab. In case of controls instead of BsAb, anti-RBC Ab or anti-Lis Ab or mixture of the two was added. **A**) RBC antigen was coated and anti-Lis mAb was used for detection. mBsAb was employed. **B**) Listeria Ag coated and anti-RBC mAb detected the signal. mBsAb was used. **C**) RBC antigen was coated and anti-Lis pAb was used for detection. pBsAb was exploited. **D**) Listeria Ag coated and anti-RBC pAb detected the signal. pBsAb was used. Only BsAbs could give significant signals. On the other hand, the controls gave very feeble signals validating the formation of BsAb. At least three independent experiments were performed and data are represented as mean of at three independent experiments ± SD value. (*P* values; BsAb vs anti-RBC control <0.05, BsAb vs anti-Lis control <0.05, BsAb vs anti-RBC and anti-Lis mixture <0.05).

Attempts were made to use increasing concentrations (0–60 mM) of reducing agents, viz. β–mercaptoethanesulfonic acid sodium salt and β–mercaptoethanol to reduce the disulphide bonds of antibodies (data not shown for β–mercaptoethanol). It was found that β–mercaptoethanesulfonic acid sodium salt (60 mM) as well as β–mercaptoethanol (50 mM) were equally efficient in the formation of the monovalent antibody fragment (data not shown for β–mercaptoethanol). A pilot study suggested that the reduction and re-oxidation of the mixture of monovalent antibody from two different sources (anti-human erythrocyte and anti-*Listeria* cell surface antigens) led to the formation of bispecific antibody. As shown in **Fig. 3A**, 60 mM of β–mercaptoethanesulfonic acid sodium salt caused the optimum reduction of disulphide linkages of both anti-RBC and anti-*Listeria* antibodies of monoclonal origin. Similar to monoclonal antibodies, 60 mM of β–mercaptoethanesulfonic acid sodium salt redendered efficient reduction of antibodies of polyclonal nature to mvAbs (**Fig. S1A**). The controlled re-oxidation of treated antibodies led to the generation of BsAbs (**Fig. 3B and S** **1B**). The affinity purified BsAbs were evaluated for their potential to bind RBC cell surface as well as *Listeria monocytogenes* cell surface proteins employing ELISA. Fig. 4 reveals that upon coating either of the two antigens viz. RBCs and *Listeria* Ag followed by incubation with BsAb and and detecting the signal using the other antigen exhibited a significantly higher binding of BsAb to antigen as compared to controls (*P*<0.05; for mBsAb or pBsAb vs control) indicating the functional specificity of the BsAbs prepared in-house using monoclonal as well as polyclonal antibodies. Interaction of coated RBC surface antigen occupied one arm of the mBsAb, keeping other arm still free to react with *Listeria* antigen incubated in the next step. Presence of BsAb bound *Listeria* antigen recognizes *Listeria* specific antibody raised in rabbit. ELISA results show development of colour upon exposure with HRP conjugated goat anti rabbit antibodies. The ELISA result also show that incubation with anti-listeria or anti-RBC and even mixture of anti-RBC and anti-listeria do not give colour signal when used in the same ELISA format.

**Figure 5:**
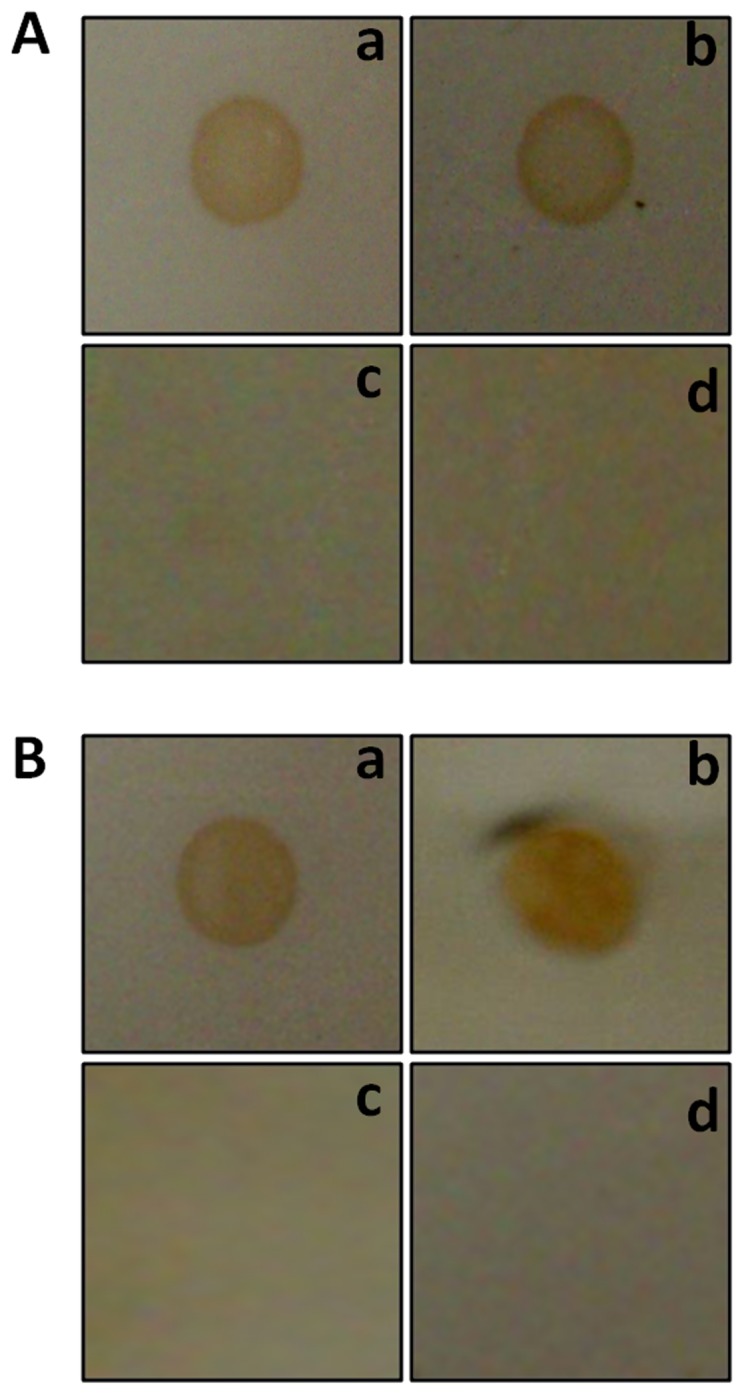
Dot blot analysis. **A**) **a** and **b** show recognition of *Listeria* surface antigen and RBC ghost proteins by mBsAb respectively. **c** and **d** exhibit controls where PVDF membrane was coated with *E. coli* and *Salmonella* Ags respectively and allowed to react with mBsAb (specific to *Listeria monocytogenes* and RBCs). A negative result was obtained in controls. **B**) **a** and **b** exhibit binding of pBsAb to Listeria surface protein and RBC membrane proteins respectively. PVDF membrane coated with *E. coli* and *Salmonella* Ags shows negative result when allowed to react with pBsAb (**c** and **d** respectively). At least three independent experiments were performed for each sample and data are representative of three independent experiments with similar observations.

**Figure 6:**
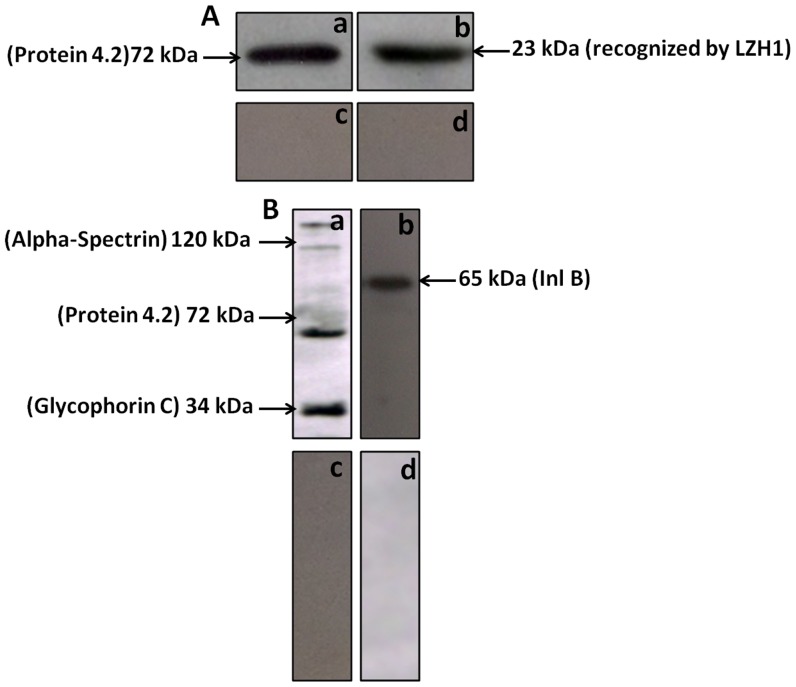
Western blot analysis. **A**) **a** and **b** show the reactivity of mBsAb to RBC ghost proteins and *Listeria* surface protein respectively while **c** and **d** exhibit incapability of mBsAb to react with *E. coli* and *Salmonella* Ags respectively. Similarly,pBsAb reacted well with RBC ghost proteins and *Listeria* InlB protein (**Ba** and **Bb** respectively). On the other hand negative result is observed for *E. coli* and *Salmonella* proteins when pBsAb is used for recognition (**Bc** and **Bd** respectively). At least three independent experiments were performed for each sample and data are representative of three independent experiments with similar observations.

In the next set of experiment where *Listeria* surface antigen coated ELISA plate was incubated with mBsAb already exposed RBC surface antigen. The plate was next coated with rabbit anti RBC surface antigen specific antibodies. After stipulated washing steps followed by incubation with HRP coated goat anti rabbit antibody reacted with *Listeria* coated wells in concentration dependent manner. In a manner similar to the result shown in Fig 3A the mixture of antibodies specific for *Listeria* as well as RBC antigen does not give positive signal. The negative controls consisting of anti-listeria or anti-RBC surface specific antibodies also do not give positive signal. Dot blot and Western blot analyses further confirmed the specificity of BsAbs for InlB as well as RBC antigens (Fig. 5 and Fig. 6). As shown in Fig. 5, mBsAbs as well as pBsAbs did not interact with *E. coli* and *Salmonella* antigens. On, the contrary, they reacted explicitly with *Listeria* antigen InlB and RBC antigens. Similar results were observed for Western blot (Fig. 6) which also showed reactivity of BsAbs (mBsAbs as well as pBsAbs) only with InlB and RBC antigens. No reactivity could be seen with control antigens.

### Interaction of RBCs and *Listeria monocytogenes* cells in the presence of BsAb

**Figure 7:**
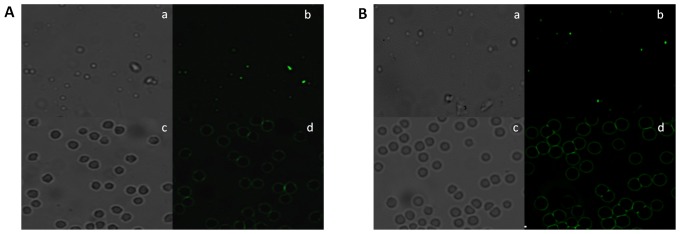
BsAb specificity for Listeria and RBC antigens as revealed by Fluorescence Microscopy. **A**. mBsAb interaction with cell surface proteins of *Listeria monocytogenes* (**a and b**), mBsAb binding capacity to human RBCs (**c and d**). **B**. Affinity of pBsAb with target surface molecules of *Listeria monocytogenes* cells (**a and b**) and RBCs (**c and d**). At least three independent experiments were performed for each sample and data are representative of three independent experiments with similar observations.

**Figure 8:**
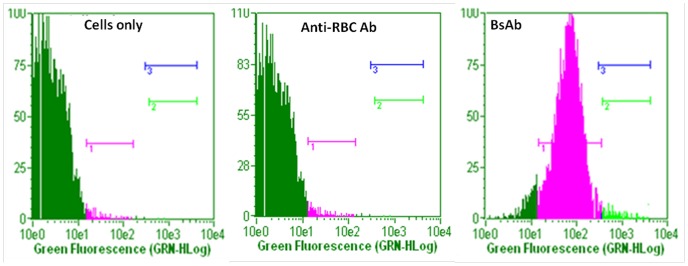
Interaction of *Listeria monocytogenes* cells with BsAb employing fluorescence-activated cell sorting analysis. The cells with log fluorescence intensities were gated BsAb and *Listeria* cells. (**a**) *Listeria monocytogenes* cells only, (**b**) Anti-RBC Ab incubated with *L. monocytogenes* cells (**c**) Bispecific antibody of polyclonal origin (pBsAb) incubated with *Listeria* cells for 1 hr. At least three independent experiments were performed for each sample and data are representative of three independent experiments with similar observations.

The functional specificity of BsAbs was also ascertained by fluorescence microscopy and fluorescence-activated cell sorting (FACS) analysis. The resulting agglutinated mass comprising erythrocytes and *L. monocytogenes* was visualized by fluorescence microscopy employing FITC labeled anti-rabbit IgGs. RBCs and *L. monocytogenes* cells were incubated with mBsAb as well as pBsAb and visualized by fluorescence microscopy employing FITC labeled anti-mouse IgGs and anti-rabbit IgGs respectively. Figure 7 shows fluorescence restricted to the surface of erythrocytes and *Listeria* cells enumerating binding of BsAbs to the cell surfaces. FACS analysis further confirmed the ability of pBsAbs to recognize *L. monocytogenes* cells specifically in contrast to control anti-RBC antibodies that failed to react with *Listeria monocytogenes* (Figure 8). As the incubation period of pBsAbs with *Listeria* increased from 1 hr to 2 hrs, an augmentation of signal was observed enumerating enhanced binding of BsAb to Listeria cells with time. A negligible signal was observed for Listeria cells either incubated alone or with anti-RBC antibodies. Similarly, in-house prepared BsAbs did not recognize unrelated organisms such as *E. coli* and *Salmonella typhimurium* (data not shown).

### Detection of *Listeria monocytogenes* cells by agglutination assay and its sensitivity determination

**Figure 9:**
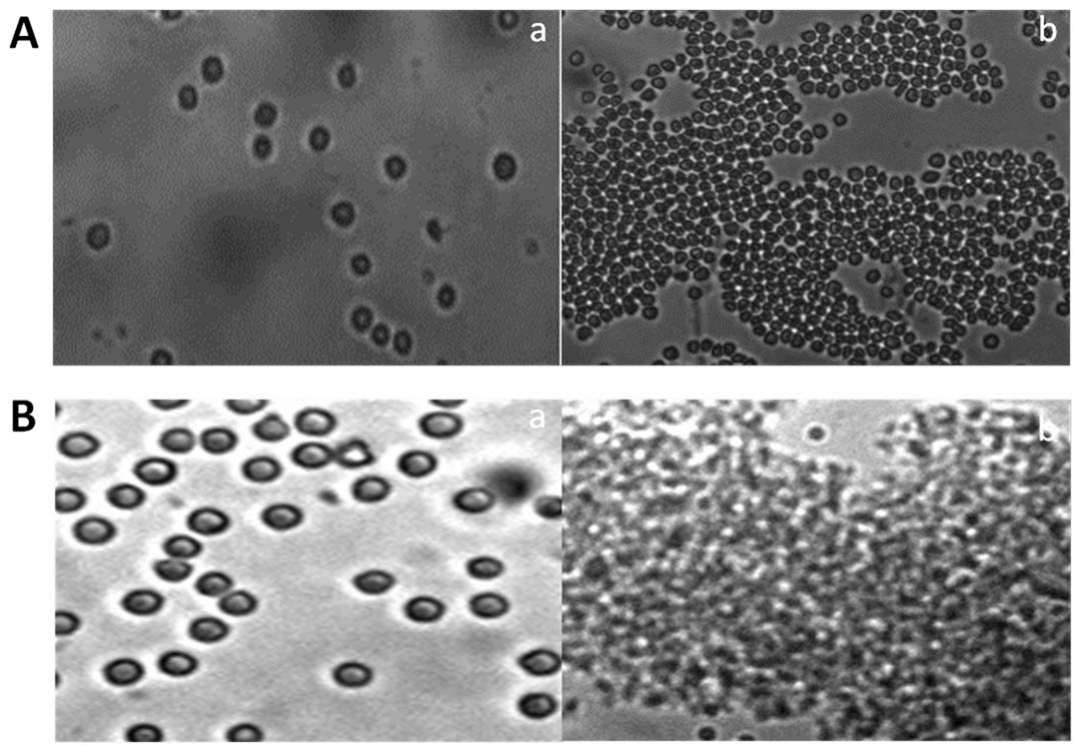
Bispecific antibody mediated haemagglutination. mBsAb (**A**) as well as pBsAb (**B**) rendered agglutination of RBCs and *Listeria* cells on a glass slide. A given volume (50 μl of 200 ng/ml) of BsAb specific against both RBCs and *Listeria* cell surface Ag was mixed with *Listeria* cells-negative (**Aa, Ba**) or *Listeria* cells-positive (**Ab, Bb**). After 1 hr of incubation, the RBC agglutination results were observed under a microscope (right panel; magnification, 20X). At least three independent experiments were performed for each sample and data are representative of three independent experiments with similar observations.

**Figure 10:**
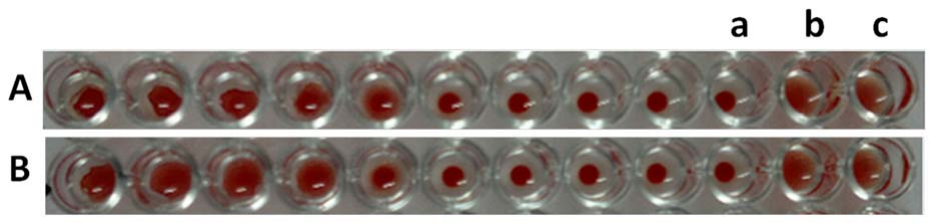
Demonstration of hemagglutination employing mBsAb (A) and pBsAb (B) against human red blood cells and *Listeria* cells. The wells contain a constant number of RBC (20 μl of 60% hematocrit) and *Listeria* cells (10^3^ cells) plus serial two-fold dilutions of BsAb. The spread pattern in the experimental series indicates positive hemagglutination through well 5 and 4 respectively for mBsAb and pBsAb incubated wells. The wells marked as a, b and c were incubated with anti-Lis Ab, anti-RBC Ab and a mixture of these two respectively. At least three independent experiments were performed for each sample and data are representative of three independent experiments with similar observations.

**Figure 11:**
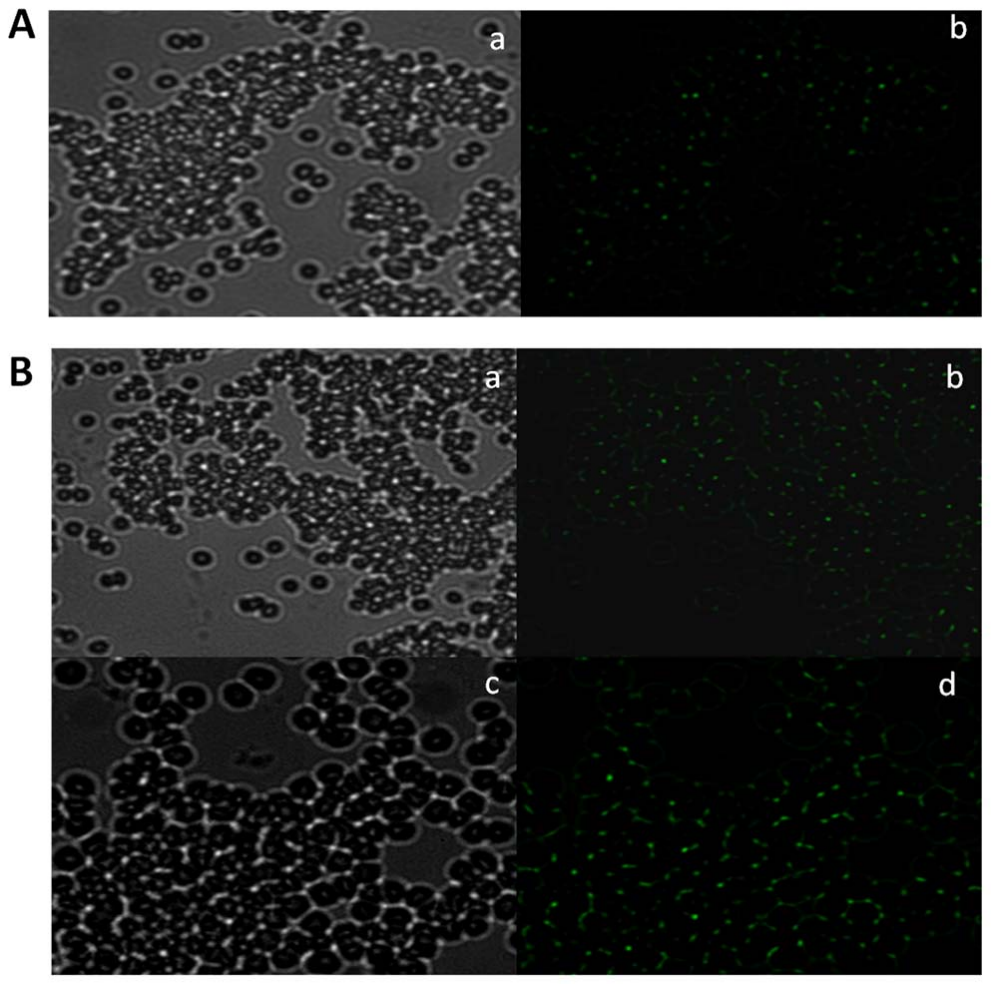
Fluorescence microscopy demonstrating RBC and *Listeria* cells agglutination in the presence of mBsAb (A) and pBsAb (B) generated for simultaneous interaction with both RBC as well *Listeria* cell surface antigen. Phase contrast (Aa and Ba) and fluorescence images (Ab and Bb) at x20. Same as Ba and Bb at x40 (Bc and Bd). At least three independent experiments were performed for each sample and data are representative of three independent experiments with similar observations.

To establish further that both the anti-RBC and the anti-*Listeria* antigen binding sites were located on the two arms of the same bispecific antibody, we performed agglutination tests by incubating two fold serial dilution of mBsAbs as well as pBsAbs with the mixture of RBCs and *Listeria* cells. *Listeria monocytogenes* cells containing many copies of *Listeria* antigens (cf. InlB) can act as a bridge between two or more BsAbs and accentuate agglutination. As shown in figure 9, both mBsAbs and pBsAbs caused the agglutination in the presence of *Listeria* cells. The BsAbs mediated agglutination was also studied in 96 well plate formats (Figure 10). In concordance with previous result, mBsAbs as well as pBsAbs were found to be equally efficient in inducing agglutination in the presence of *Listeria* cells. Figure 11 shows fluorescence micrograph of agglutinated mass, where the BsAbs of monoclonal as well as polyclonal origins facilitating the agglutination of two cell populations (*L. monocytogenes* as well as erythrocytes) are highlighted by FITC-conjugated antibody.

**Figure 12:**
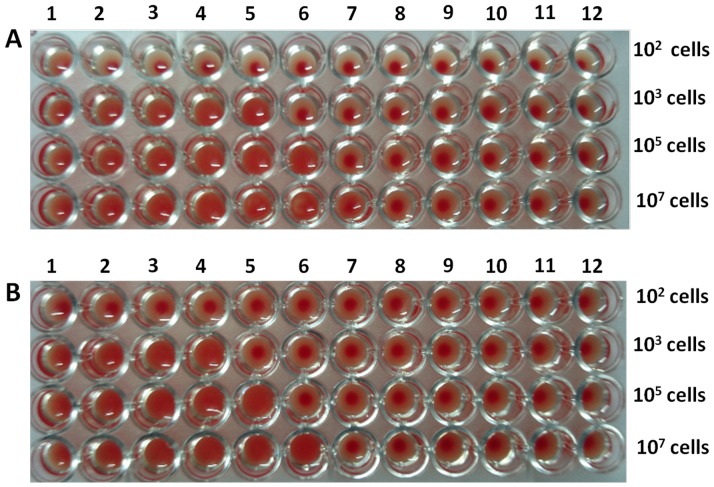
Sensitivity of agglutination assay. Various densities of *Listeria* cells (from wells 1-10) were incubated with fixed number of RBC cells in serial two-fold dilutions of BsAbs. *Listeria* cells with a density of 10^3^ or above induced agglutination of RBCs in the presence of mBsAbs (**A**) as well as pBsAbs (**B**). Wells 11 and 12 harbor *E. coli* and *S. typhimurium* cells. No agglutination is observed when these unrelated pathogens are incubated with BsAbs (mBsAbs or pBsAbs) even at densities as high as 10^7^ cells. At least three independent experiments were performed for each sample and data are representative of three independent experiments with similar observations.

Keeping into consideration the fact that to evaluate potential of BsAbs for analysis of food samples, it was necessary to check their sensitivity in agglutination assay. Various number of *Listeria* cells were incubated with a fixed density of RBCs in the presence of BsAbs (both mBsAbs and pBsAbs) to determine the sensitivity of agglutination assay. *Listeria* cells when incubated with either mBsAbs or pBsAbs at a density of at least 10^3^ cells were found to induce agglutination of the RBCs (Figure 12). The specificity of in-house prepared BsAb was established employing non-related pathogens such as *E. coli* and *S. typhimurium*. The presence of two unrelated pathogens even at very high concentration (10^7^ cells) did not induce agglutination of the erythrocyte (**Figure 12**).

### Validation of bispecific antibody based agglutination assay detection method in food samples

**Figure 13:**
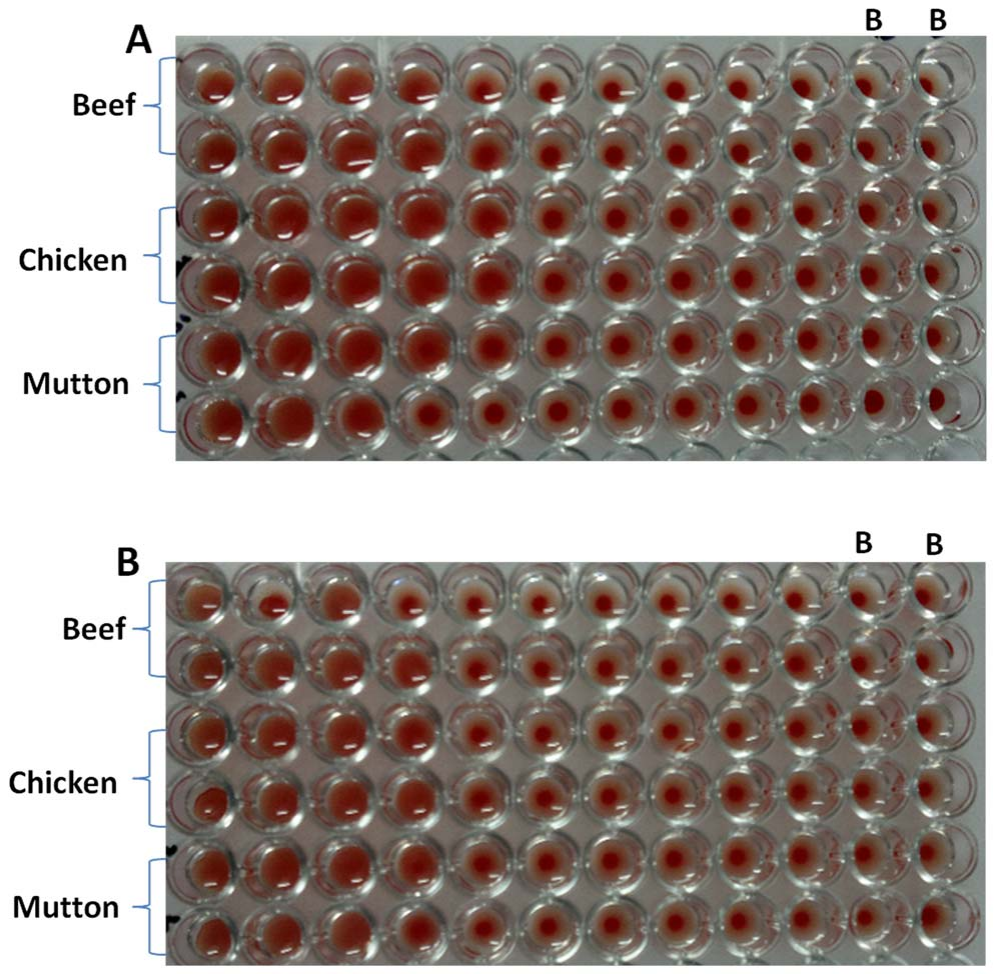
Detection of *Listeria monocytogenes* using agglutination assay in inoculated meat (beef, chicken and mutton) samples. mBsAbs as well as pBsAbs rendered agglutination of RBCs when incubated with *Listeria* inoculated meat. The wells marked as ‘B’ are controls lacking *Listeria* cells. At least three independent experiments were performed for each sample and data are representative of three independent experiments with similar observations.

As the ultimate purpose of the development of bispecific antibodies was to use it for the detection of *L. monocytogenes* in food, it is obligatory to validate its performance in food samples. The commercially available food samples were confirmed to be free of detectable pathogens. Therefore, testing the agglutination assay in food was carried out by artificially inoculating with *L. monocytogenes* meat (beef, mutton and chicken) samples. The bispecific antibodies were found to be capable of agglutinating the blood when incubated with artificially contaminated food samples (Figure 13).

## Discussion

Infections and infectious diseases continue to plague human population globally inspite of the improved hygiene, sanitation and availability of a spectrum of antibiotics. Annually, a quarter of population in US and ∼20% population in UK are estimated to be affected by food-borne diseases [28]. There have been multiple outbreaks caused by *Escherichia coli* O157:H7 and *Salmonella* respectively in US in years 2006 and 2007. The *Escherichia coli* O157:H7 outbreaks have been associated with green leafy vegetables like spinach and lettuce and a total of 199 people were infected in 26 states by consuming contaminated spinach, out of which 3 succumbed to the infection [29]. A *Salmonella* outbreak spread through tomatoes and peanut butter caused severe illness in 288 individuals [28].

Data from developing/poor countries are fragmentary but that available show that at any given time millions suffer from bacterial infections or are under constant threat. Illiteracy, poverty and lack of access to hygiene, healthy and uninfected food can aggravate the problem. Besides causing food poisoning, food-borne pathogens can evoke other disease manifestations like stomach ulcers (eg. *Helicobacter pylori*), tuberculosis (*Mycobacterium tuberculosis*), meningitis (species of *Streptococci, Neisseria*), cholera (*Vibrio cholerae*), sexually transmitted diseases, and nosocomial infections [30]. The rapid detection of pathogens and other microbial contaminants in food is therefore highly critical for ensuring the safety of consumers.

The widespread occurrence of food-borne infections combined with apparent lack of simple and rapid bio-sensing technique persuaded us to develop an assay that can detect the presence of pathogen specifically with naked eye. We were successful in developing a simple strategy to rapidly but accurately identifying the presence of *Listeria* without the need of any sophisticated instrumentation. The agglutination of erythrocytes, which is induced by a bifunctional antibody recognizing surface antigen of *Listeria* and erythrocytes only in the presence of *Listeria* cells could be visualized by the naked eye in 10-30 minutes. To the best of our knowledge, this is the first report employing erythrocyte surface specific bi-specific antibodies to screen the presence of pathogens in sample foods and beverages.

The following can be inferred from the data of the present study:

- Redox method reported in the present study causes reduction of parent antibodies leading to formation of monovalent population. The oxidation mediated reassociation of light & heavy chain leads to formation of BsAb that has dual specificity for both erythrocyte as well as *Listeria* surface protein simultaneously.
- The in-house developed BsAb antibody can recognize both isolated (free) as well as cell surface bound antigen (*Listeria* surface antigen InlB) of *Listeria monocytogenes* with one arm while other arm can interact with erythrocyte surface antigen.
  - The co-incubation of erythrocyte as well as *Listeria* (whole bacteria) in the presence of BsAb ensues in agglutination of RBC as well as *Listeria monocytogenes*.
  - The interaction of BsAb is specific as no agglutination takes place in the presence of unrelated food borne bacteria such as *E. coli* or *Salmonella typhimurium*.
  - Erythrocyte agglutination is rapid and sensitive and can be seen with both microscope as well as naked eyes. The agglutination can take place in the presence of 10^2^–10^3^ *Listeria* cells.

Once formation of BsAb from monoclonal antibodies (mouse monoclonal anti-*Listeria* LZH1 IgG1 and mouse monoclonal IgG2a raised against the human erythrocyte membrane protein (Protein4.2 [2G-12]) was established by Western blot analysis (Figure 5), we were encouraged to construct BsAb of polyclonal origin. Remarkably, it was also possible to prepare the BsAbs with antibodies raised against intact red blood cells and InlB, an indispensible protein of *Listeria*. Specific agglutination could be induced by the BsAb constructed from either monoclonal or polyclonal antibodies. The use of antibodies recognizing InlB, an important surface antigen of virulent *Listeria* makes this approach highly cost effective and addresses the risk of food sample contamination with such important bacteria. We prefer to use pBsAb over mBsAb as the latter does not jeopardizes loss of recognition of single surface molecule in the mutated forms. Reports that *Listeria monocytogenes* isolate can undergo a point mutation that abolishes the recognition by a monoclonal antibody is available [30].

The specificity of the BsAbs constructed from monoclonal as well as polyclonal antibodies was ascertained by conventional assays including ELISA, dot blot and Western blot analysis but agglutination assay was the center stage experimental strategy. This obviously overcame some of the limitations of ELISA, Western blot or dot blot assays. Antibodies were raised against RBC ghost and *L. monocytogenes* membrane protein (Inl B) as revealed by ELISA, dot blot assay and fluorescence microscopy (**Figure 2**). The ELISA result shown in **Figure 4** confirms the formation of bispecific antibody as depicted by comparatively increased absorbance in response to BsAb binding with respect to controls. The generated bispecific antibody could effectively bind RBC ghosts and the surface antigens of *L. monocytogenes* was confirmed by dot blot and Western blot analyses as revealed in **Figure 5** and **Figure 6** respectively and cell surface restricted fluorescence as depicted by fluorescence microscopic image (**Figure 7**). The potential of the constructed bispecific antibody, to recognize populations of *L. monocytogenes* was further confirmed by fluorescence activated cell sorting analysis as well (**Figure 8**).

**Figure 14:**
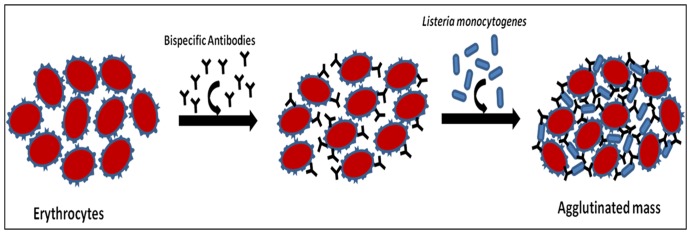
Schematic agglutination representation of erythrocytes and *Listeria monocytogenes* cells mediated by bispecific antibodies.

Interestingly, the agglutination was evident under a light microscope (**Figure 9**) and also to the naked eyes (**Figure 10**). The higher dilution of antibody does not reveal the presence of an agglutinated mass because of the decrease in the concentration of bispecific antibodies. That bispecific antibodies adhered to the agglutinated mass could be clearly seen in the fluorescence microscopic image (**Figure 11**). The agglutination assay was found to be sensitive to agglutinate cells with density more than or equal to 10^3^ cells (**Figure 12**). The BsAbs of either monoclonal or polyclonal origin failed to agglutinate RBCs in the presence of unrelated pathogens viz. *E.coli* and *S. tyhimurium* even at very high densisities (**Figure 12**) revealing the specificity of the in-house generated BsAbs. Moreover, as depicted in **figure 13**, the agglutination assay was also able to detect *L. monocytogenes* from artificially inoculated meat samples (beef, chicken and mutton) although the commercially procured food samples were found to be free from bacterial contamination (data not shown). A schematic representation of the agglutination process employing bispecific antibodies capable of recognizing RBCs and *Listeria monocytogenes* is depicted in **Figure 14**.

Finally, in the present report, we describe the construction of BsAb based biosensor for the detection of *L. monocytogenes* employing either monoclonal antibodies specific to human erythrocyte membrane protein antigen (Protein 4.2 (2G-12)) or *Listeria monocytogenes* surface antigen, or polyclonal antibodies recognizing surface structures present on two cell populations. The use of polyclonal antibodies recognizing more than one surface antigen, makes production of BsAbs simpler and convenient. The remarkable specificity of the technique employed is evident from the absence of cross-reactivity of the in-house prepared BsAbs with other pathogens such as *E. coli* and *S. typhimurium*. The strategy is remarkably cost effective as it does not necessitate any sophisticated instrumentation or expensive chemicals. The rapid onset of agglutination reaction visible to the naked eyes makes it highly suitable for maintaining the quality control over food supply in various eatery outlets.

## Conclusion

We describe in this report the successful application of a bispecific antibody for the rapid and specific detection of *Listeria* in food and other biological samples. The BsAbs induced visible agglutination of the RBCs in the presence of *L. monocytogenes* in less than an hour. The simple and rapid agglutination assay was highly specific and effective in sensing the presence of the pathogen in the test samples. The present report offers a platform technique with multiple potential applications, including the construction of bioassay / biosensor devices for the detection of *Listeria monocytogenes*, and the technology may well be modified appropriately for the detection of any pathogen. The technique is so simple and inexpensive that it is adaptable for the screening of food samples for a variety of pathogen even in households and restaurant setups.

## Supporting Information

Figure S1 **Formation of pBsAb employing Redox method.** Non-Reducing SDS-PAGE analysis of mvAb and BsAb of polyclonal origin. A range of β-mercaptoethanesulphonic acid concentrations were analysed for effective reduction polyclonal anti-erythrocyte antibodies. β-mercaptoethanesulphonic acid concentration of 60 mM (reducing condition) efficiently cleaved the inter- disulphide bridges between heavy chains and lead to formation of mvAb (≥75 kD) of polyclonal anti-RBC antibodies (**A**). Similarly, 60 mM of β-mercaptoethanesulphonic also rendered effective reduction of polyclonal anti-*Listeria* antibodies (data not shown). Dialysis in PBS (oxidizing condition) resulted in reformation of mvAb against RBC and *Listeria* into pBsAb (**B**). The lanes marked pAb, red., oxid. and M in **B** enumerate anti-Listeria polyclonal antibody, reduction of anti-Listeria pAb using 60 mM of β-mercaptoethanesulphonic acid, oxidation of anti-RBC mvAb and anti-Listeria mvAb and protein marker respectively. H_2_L_2_ =  whole antibody, HL = mvAb, H = heavy chain, red. = reducing condition, oxid. = oxidizing condition.
(TIF)

## References

[1] Holliger P, Hudson PJ (2005) Engineered antibody fragments and the rise of single domains. Nat Biotechnol 23: 1126–1136. 16151406 10.1038/nbt1142

[2] Zhang J, Tanha J, Hirama T, Khieu NH, To R, et al (2004) Pentamerization of single-domain antibodies from phage libraries: a novel strategy for the rapid generation of high-avidity antibody reagents. J Mol Biol 335: 49–56. 14659739 10.1016/j.jmb.2003.09.034

[3] Hussack G, Luo Y, Veldhuis L, Hall JC, Tanha J, et al (2009) Multivalent anchoring and oriented display of single-domain antibodies on cellulose. Sensors 9: 5351–5367. 22346702 10.3390/s90705351 PMC3274147

[4] De Jonge J, Heirman C, de Veerman M, van Meirvenne S, Moser M, et al (1998) In vivo retargeting of T cell effector function by recombinant bispecific single chain Fv (anti-CD3 x anti-idiotype) induces long-term survival in the murine BCL1 lymphoma model. J Immunol 161: 1454–1461. 9686611

[5] Carlring J, Leenheer ED, Heath AW (2011) A novel redox method for rapid production of functional bi-specific antibodies for use in early pilot studies. PLoS ONE 6: e22533. 21811628 10.1371/journal.pone.0022533 PMC3141073

[6] Menard S, Canevari S, Colnaghi MI (1989) Hybrid antibodies in cancer diagnosis and therapy. Int J Biol Markers 4: 131–134. 2693537 10.1177/172460088900400301

[7] Suresh MR, Cuello AC, Milstein C (1986) Bispecific monoclonal antibodies from hybrid hybridomas. Methods Enzymol 121: 210–228. 3724461 10.1016/0076-6879(86)21019-8

[8] Lu D, Zhang H, Koo H, Tonra J, Balderes P, et al (2005) A fully human recombinant IgG-like bispecific antibody to both the epidermal growth factor receptor and the insulin-like growth factor receptor for enhanced antitumor activity. J Biol Chem 280: 19665–19672. 15757893 10.1074/jbc.M500815200

[9] Orcutt KD, Ackerman ME, Cieslewicz M, Quiroz E, Slusarczyk AL, et al (2010) A modular IgG-scFv bispecific antibody topology. Protein Eng Des Sel 23: 221–228. 20019028 10.1093/protein/gzp077 PMC2841541

[10] Shen J, Vil MD, Jimenez X, Iacolina M, Zhang H, et al (2006) Single variable domain-IgG fusion. A novel recombinant approach to Fc domain-containing bispecific antibodies. J Biol Chem 281: 10706–10714. 16481314 10.1074/jbc.M513415200

[11] Wu C, Ying H, Grinnell C, Bryant S, Miller R, et al (2007) Simultaneous targeting of multiple disease mediators by a dual-variable-domain immunoglobulin. Nat Biotechnol 25: 1290–1297. 17934452 10.1038/nbt1345

[12] Hamon M, Bierne H, Cossart P (2006) *Listeria monocytogenes*: a multifaceted model. Nat Rev Microbiol 4: 423–434. 16710323 10.1038/nrmicro1413

[13] Guttikonda S, Wang W, Suresh M (2004) Molecular zipper assays: a simple homosandwich with the sensitivity of PCR. J Pharm Pharmaceut Sci 7: 7–16. 15850543

[14] Allos BM, Moore MR, Griffin PM, Tauxe RV (2004) Surveillance for sporadic foodborne disease in the 21st century: the FoodNet perspective. Clin Infect Dis 38: S115–S120. 15095179 10.1086/381577

[15] Padmapriya P, Banada, Bhunia AK (2008) Antibodies and Immunoassays for Detection of Bacterial Pathogens. Principles of bacterial detection: biosensors, recognition receptors and Microsystems III: 567–602.

[16] Hamon M, Bierne H, Cossart P (2006) Listeria monocytogenes: a multifaceted model. Nat Rev Microbiol 4(6): 423–434. 16710323 10.1038/nrmicro1413

[17] Gasanov U, Hughes D, Hansbro PM (2005) Methods for the isolation and identification of *Listeria* spp. and *Listeria monocytogenes*: A review. FEMS Microbiol Rev 29: 851–875. 16219509 10.1016/j.femsre.2004.12.002

[18] Dodge JT, Mitchell C, Hanahan DJ (1963) The preparation and chemical characteristics of haemoglobin free ghost of human erythrocytes. Arch Biochem Biophys 100: 119–130. 14028302 10.1016/0003-9861(63)90042-0

[19] Iglesias BF, Catala A (2005) Rat, equine and bovine erythrocyte ghosts exposed to t-butyl hydroperoxide as a model to study lipid peroxidation using a chemiluminescence assay. Res Vet Sci 79: 19–27. 15894020 10.1016/j.rvsc.2004.10.004

[20] Mujahid S, Pechan T, Wang C (2007) Improved solubilisation of surface proteins from Listeria monocytogenes for 2-DE. Electrophoresis 28: 3998–4007. 17922522 10.1002/elps.200600858

[21] Müller S, Hain T, Pashalidis P, Lingnau A, Domann E, et al (1998) Purification of the inlB gene product of Listeria monocytogenes and demonstration of its biological activity. Infect Immun. 66(7): 3128–33. 10.1128/iai.66.7.3128-3133.1998 PMC108323 9632576

[22] Likhite N, Warawdekar UM (2011) A Unique Method for Isolation and Solubilization of Proteins after Extraction of RNA from Tumor Tissue Using Trizol. J Biomol Tech 22: 37–44. 21455480 PMC3059540

[23] Bratcher RL, Chong CA, Dray S (1974) The isolation & characterization of an antisheep red blood cell antibody having limited heterogeneity. J Immunol 112: 1337–1346. 4205526

[24] Singhal A, Bali A, Gupta CM (1986) Antibody-mediated targeting of liposomes to erythrocytes in whole blood. Biochem Biophys Acta 880: 72–77. 3080031 10.1016/0304-4165(86)90121-2

[25] Porath J, Olin B, Granstrand B (1983) Immobilized metal affinity chromatography of serum proteins on gel immobilized group III A metal ions. Arch Biochem Biophys 225: 543–547. 6687340 10.1016/0003-9861(83)90065-6

[26] Chauhan A, Zubair S, Tufail S, Sherwani A, Sajid M, et al (2011) Fungus-mediated biological synthesis of gold nanoparticles: potential in detection of liver cancer. Int J Nanomedicine 6: 2305–2319. 22072868 10.2147/IJN.S23195 PMC3205127

[27] Ohk SH, Koo OK, Sen T, Yamamoto CM, Bhunia AK (2010) Antibody-aptamer functionalized fibre-optic biosensor for specific detection of Listeria monocytogenes from food. J Appl Microbiol 109: 808–17. 20337767 10.1111/j.1365-2672.2010.04709.x

[28] Kendall PA, Hillers VV, Medeiros LC (2006) Food safety guidance for older adults. Clin Infect Dis 42: 1298–1304. 16586390 10.1086/503262

[29] CDC. Update on multi-state outbreak of E. coli O157:H7 infections from fresh spinach. (2006) In: Department of Health and Human Services (ed) E. coli O157:H7 Outbreak in Spinach. Center for Disease control and Prevention (CDC), Atlanta, Georgia. Available: http://www.cdc.gov/foodborne/ecolispinach/100606.htm.

[30] Bhunia AK (2006). Detection of significant bacterial pathogens and toxins of interest in homeland security. In: Amass SF, Bhunia AK, Chaturvedi AR, Dolk DR, Peeta S, and Atallah MJ, (eds) The Science of Homeland Security. Purdue University Press, West Lafayette, Indiana. pp. 109–149.

