# Supplementary figures and images for: An Alternative Chemical Redox Method for the Production of Bispecific Antibodies: Implication in Rapid Detection of Food Borne Pathogens
Source: PLoS One. 2014 Mar 17;9(3):e91255. doi: 10.1371/journal.pone.0091255 (PMC3956663; doi:10.1371/journal.pone.0091255)

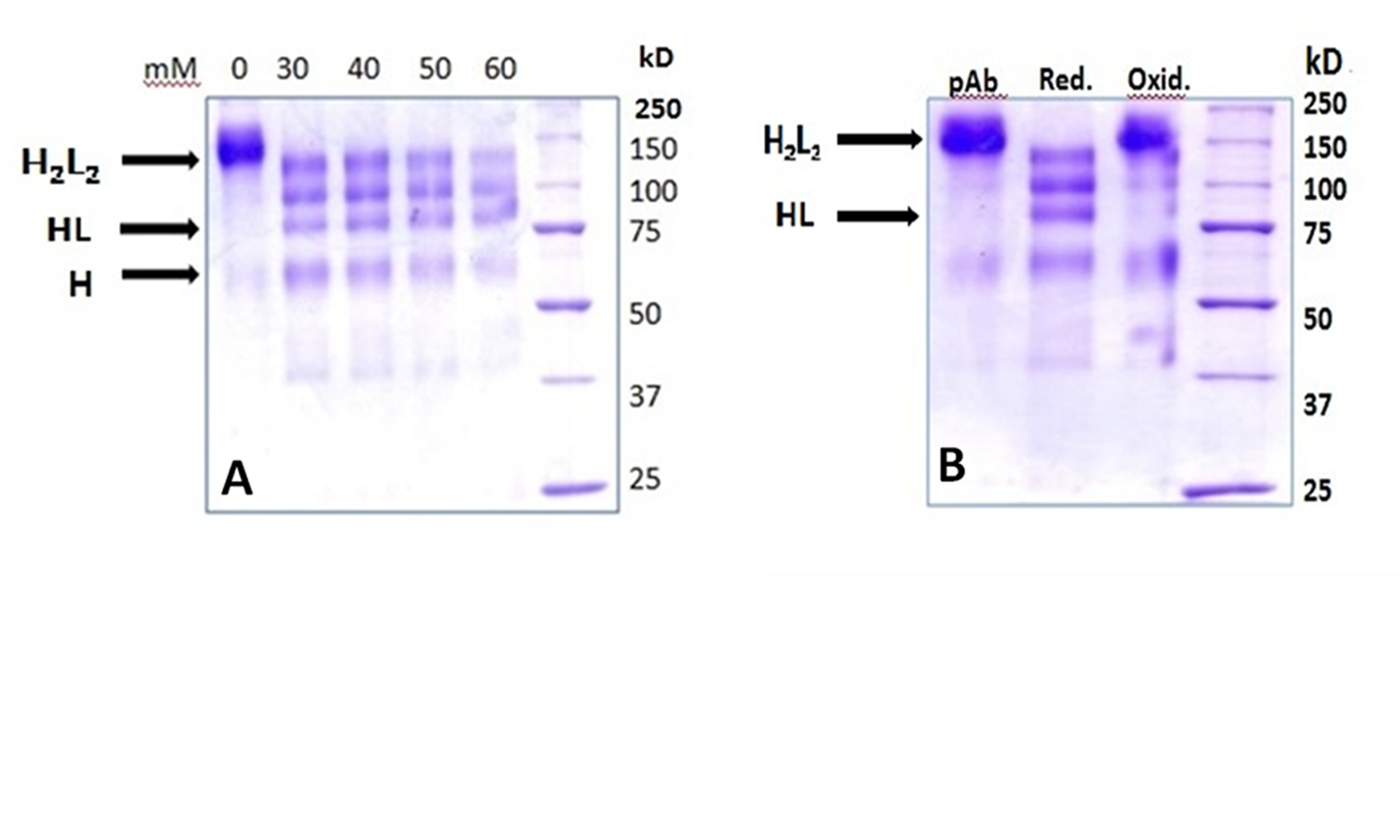

Supplement: Figure S1 — Formation of pBsAb employing Redox method. Non-Reducing SDS-PAGE analysis of mvAb and BsAb of polyclonal origin. A range of β-mercaptoethanesulphonic acid concentrations were analysed for effective reduction polyclonal anti-erythrocyte antibodies. β-mercaptoethanesulphonic acid concentration of 60 mM (reducing condition) efficiently cleaved the inter- disulphide bridges between heavy chains and lead to formation of mvAb (≥75 kD) of polyclonal anti-RBC antibodies (A). Similarly, 60 mM of β-mercaptoethanesulphonic also rendered effective reduction of polyclonal anti-Listeria antibodies (data not shown). Dialysis in PBS (oxidizing condition) resulted in reformation of mvAb against RBC and Listeria into pBsAb (B). The lanes marked pAb, red., oxid. and M in B enumerate anti-Listeria polyclonal antibody, reduction of anti-Listeria pAb using 60 mM of β-mercaptoethanesulphonic acid, oxidation of anti-RBC mvAb and anti-Listeria mvAb and protein marker respectively. H2L2 = whole antibody, HL = mvAb, H = heavy chain, red. = reducing condition, oxid. = oxidizing condition. (TIF) [file pone.0091255.s001.tif]
